# Multiple pericardial hematomas: a case report and mini-review in multimodality imaging

**DOI:** 10.1186/s12880-021-00617-0

**Published:** 2021-05-18

**Authors:** Aninka Saboe, Ferdy Sanjaya, Raden Erwin Affandi Soeriadi, Euis Maryani, Nuraini Yasmin Kusumawardhani, Charlotte Johanna Cool, Astri Astuti

**Affiliations:** 1 Department of Cardiology and Vascular Medicine, Faculty of Medicine, Universitas Padjadjaran – Hasan Sadikin General Hospital, Jl. Pasteur No. 38, Bandung, 40161 Indonesia; 2 Department of Nuclear Medicine and Molecular Imaging, Faculty of Medicine, Universitas Padjadjaran – Hasan Sadikin General Hospital, Bandung, Indonesia; 3 Cardiothoracic Surgery Division, Department of Surgery, Faculty of Medicine, Universitas Padjadjaran – Hasan Sadikin General Hospital, Bandung, Indonesia; 4 Cardiovascular Imaging Division, Department of Cardiology and Vascular Medicine, Faculty of Medicine, Universitas Padjadjaran – Hasan Sadikin General Hospital, Bandung, Indonesia

**Keywords:** Diagnosis, Pericardial mass, Pericardial hematoma, Multi-modality cardiac imaging, Diagnostic investigation

## Abstract

**Background:**

Pericardial hematoma is blood accumulation in the pericardial space. Although rare, it could arise in various conditions, such as after cardiac surgery. Clinical diagnosis of pericardial hematoma is implausible; thus, cardiac imaging plays a pivotal role in identifying this condition. We presented a case of multiple pericardial hematomas, which was found as an incidental finding in post-cardiac surgery evaluation. We highlighted the diagnostic challenge and the key features of multi-modality cardiac imaging in pericardial hematoma evaluation.

**Case presentation:**

An asymptomatic, 35-years old male, who underwent surgical closure of secundum atrial septal defect (ASD) one month ago, came for routine transthoracic echocardiography evaluation. An intrapericardiac hematoma was visualized at the right ventricle (RV) 's free wall side. Another mass with an indistinct border was visualized near the right atrium (RA). This mass was suspected as pericardial hematoma differential diagnosed with intracardiac thrombus. Cardiac computed tomography (CT) scan showed both masses have an attenuation of 30–40 HU; however, the mass's border at the RA side was still not clearly delineated. Mild superior vena cava (SVC) compression and multiple mediastinal lymphadenopathies were also detected. These findings are not typical for pericardial hematomas nor intracardiac thrombus; hence another additional differential diagnosis of pericardial neoplasm was considered. We pursued further cardiac imaging modalities because the patient refused to undergo an open biopsy. Single-photon emission computer tomography (SPECT)/CT with Technetium-99 m (Tc-99 m) macro-aggregated albumin (MAA) and Sestamibi showed filling defect without increased radioactivity, thus exclude the intracardiac thrombus. Cardiac magnetic resonance imaging (MRI) reveals intrapericardial masses with low intensity of T1 signal and heterogeneously high intensity on T2 signal weighted imaged and no evidence of gadolinium enhancement, which concluded the diagnosis as subacute pericardial hematomas. During follow-up, the patient remains asymptomatic, and after six months, the pericardial hematomas were resolved.

**Conclusion:**

Pericardial hematoma should be considered as a cause of pericardial masses after cardiac surgery. When imaging findings are atypical, further multi-modality cardiac imaging must be pursued to establish the diagnosis. Careful and meticulous follow-up should be considered for an asymptomatic patient with stable hemodynamic.

## Background

The incidence of pericardiac mass is rare. The differential diagnosis of intrapericardiac mass includes neoplastic and non-neoplastic lesions, such as hematoma. Pericardial hematoma is associated with various conditions and has a wide spectrum of clinical presentations, from asymptomatic to a life-threatening condition [1, 2]. Clinical diagnosis of pericardial hematoma is almost impossible; therefore, cardiac imaging is vital in diagnosis. We reported a rare case of multiple pericardial hematomas in an asymptomatic young adult with a history of open-heart surgery. We emphasized the multi-modality cardiac imaging approach and the key diagnostic features of pericardial hematoma.

## Case presentation

### Patient presentation

An asymptomatic, 35-years old male came for routine after-surgery evaluation. He had a history of successful surgical closure for secundum atrial septal defect (ASD) with a pericardial patch one month ago.

### Findings

The physical examination was unremarkable. Laboratory examinations showed haemoglobin 13 g/dL (N 14–17.4 g/dL), haematocrit 39.2% (N: 41.5–50.4%), erythrocyte 4.6 mil/uL (N 4.5–5.9 mil/uL), leukocyte 6460/uL (N 4400–11,300/uL), thrombocyte 328,000/uL (150,000–450,000/uL), prothrombin time 14.7 s (9.1–13.1 s), partial thromboplastin time 35 s (14.2–34.2 s), D-dimer 0.27 mg/L (< 0.55 mg/L), fibrinogen 245.1 mg/dL (N 238–498 mg/dL), INR 1.38 (0.8–1.2). Electrocardiography (ECG) was also within the normal limit.

### Diagnostic assessments

**Fig. 1:**
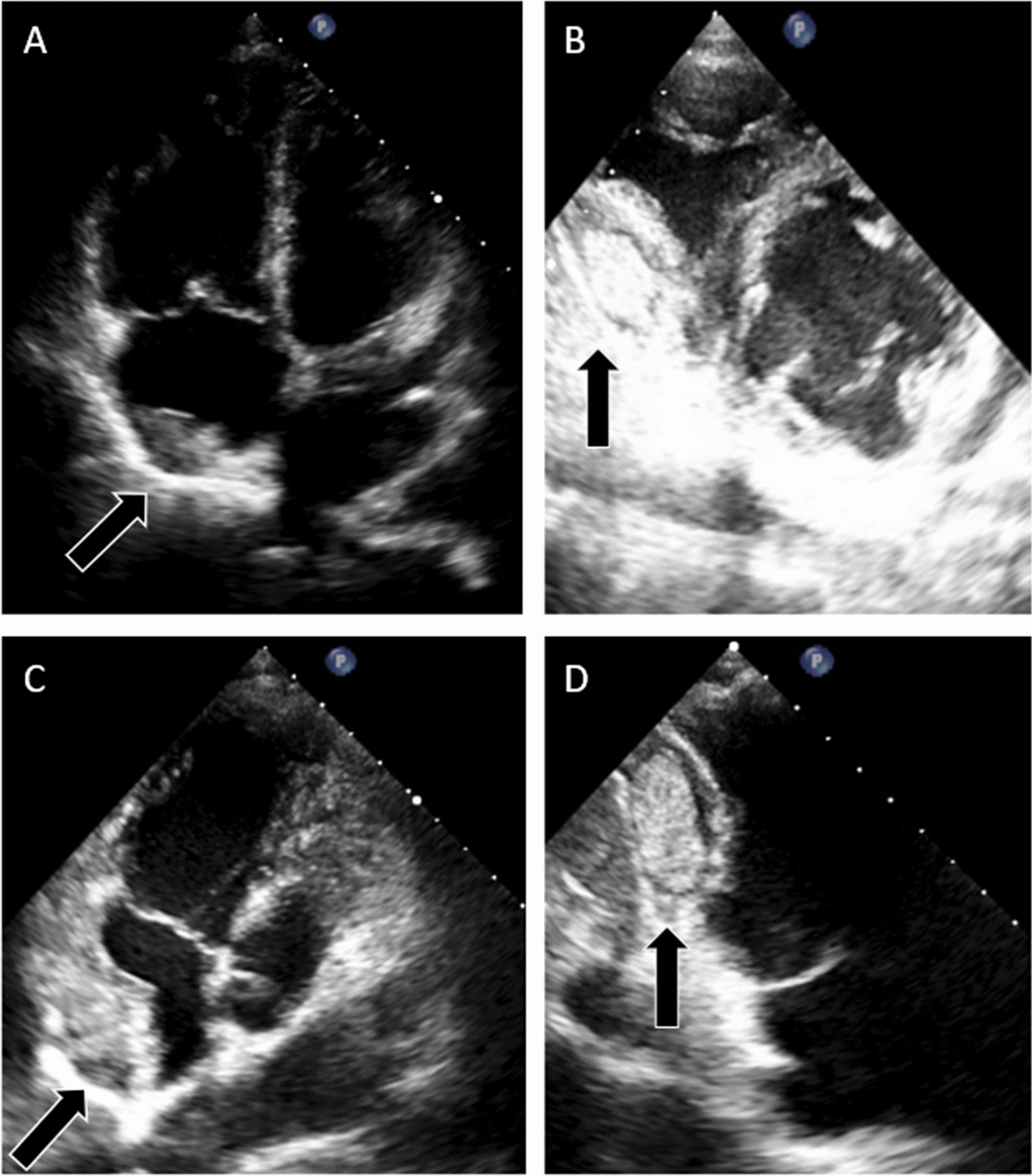
Transthoracic echocardiogram. The mass (Black Arrow) occupied the pericardial space next to the apical four-chambers view (**a**). The parasternal short-axis view (**b**) and Apical RA-focused view (**c**) revealed an indistinct border mass around the RA side. Apical RV-focused view (**d**) showed the mass near RV located within pericardial space

On Transthoracic Echocardiography (TTE), we detected two echo-dense masses (Fig. 1). An intrapericardiac hematoma with a distinct smooth rounded margin was visualized near the right ventricle's (RV) free wall. It was slightly compressing the RV chambers without any effect on the intracardiac hemodynamic. Another mass was visualized near the right atrium (RA). It had an indistinct border with the RA's free wall, which made the location was unclear. This mass was suspected as pericardial hematoma differential diagnosed with intracardiac thrombus. Despite the suspicion of RA thrombus, we did not start anticoagulation therapy because it could be fatal if the patient has pericardial hematoma. Hence, we proceed with cardiac computed tomography (CT) scan.

**Fig. 2:**
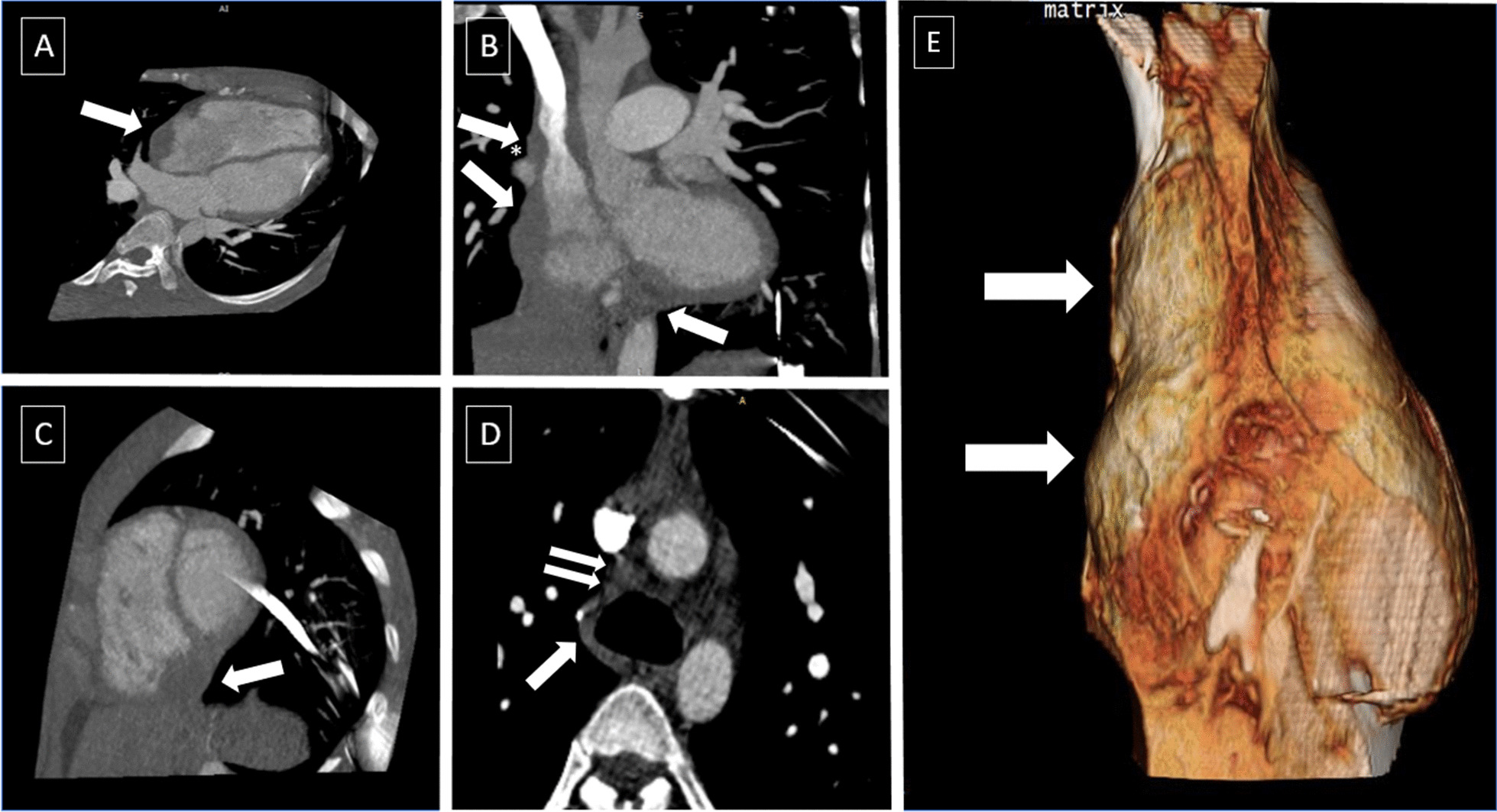
Cardiac CT scan. Cardiac CT was conducted with retrospective ECG gating technique, best diastole at 95%. 3D-multiplanar reconstructions (MPR) (A. Four chambers view, B. Coronal view, C. Short axis view) show multiple mass located near RA (**a**) and RV (**b**, **c**). The mass near RA compressing superior vena cava showed by asterisk (*). Multiple lymph node enlargements are visualized in the paratracheal region (**d**). 3D—rendered view of two masses at the right side of the heart (**e**)

Cardiac computed tomography (CT) scan showed both masses have an attenuation of 30–40 HU (Fig. 2). However, the mass's border at the RA side was still not clearly delineated. We also detected mild superior vena cava (SVC) compression without sign of obstruction. Multiple lymphadenopathies at the paratracheal region were also identified. These findings are not typical for pericardial hematomas nor intracardiac thrombus; hence another additional differential diagnosis of pericardial neoplasm was considered. After consultation with the haemato-oncologist and the cardiothoracic surgeon, an open biopsy was recommended, but the patient refused, and additional imaging was performed to pursue the diagnosis.

**Fig. 3:**
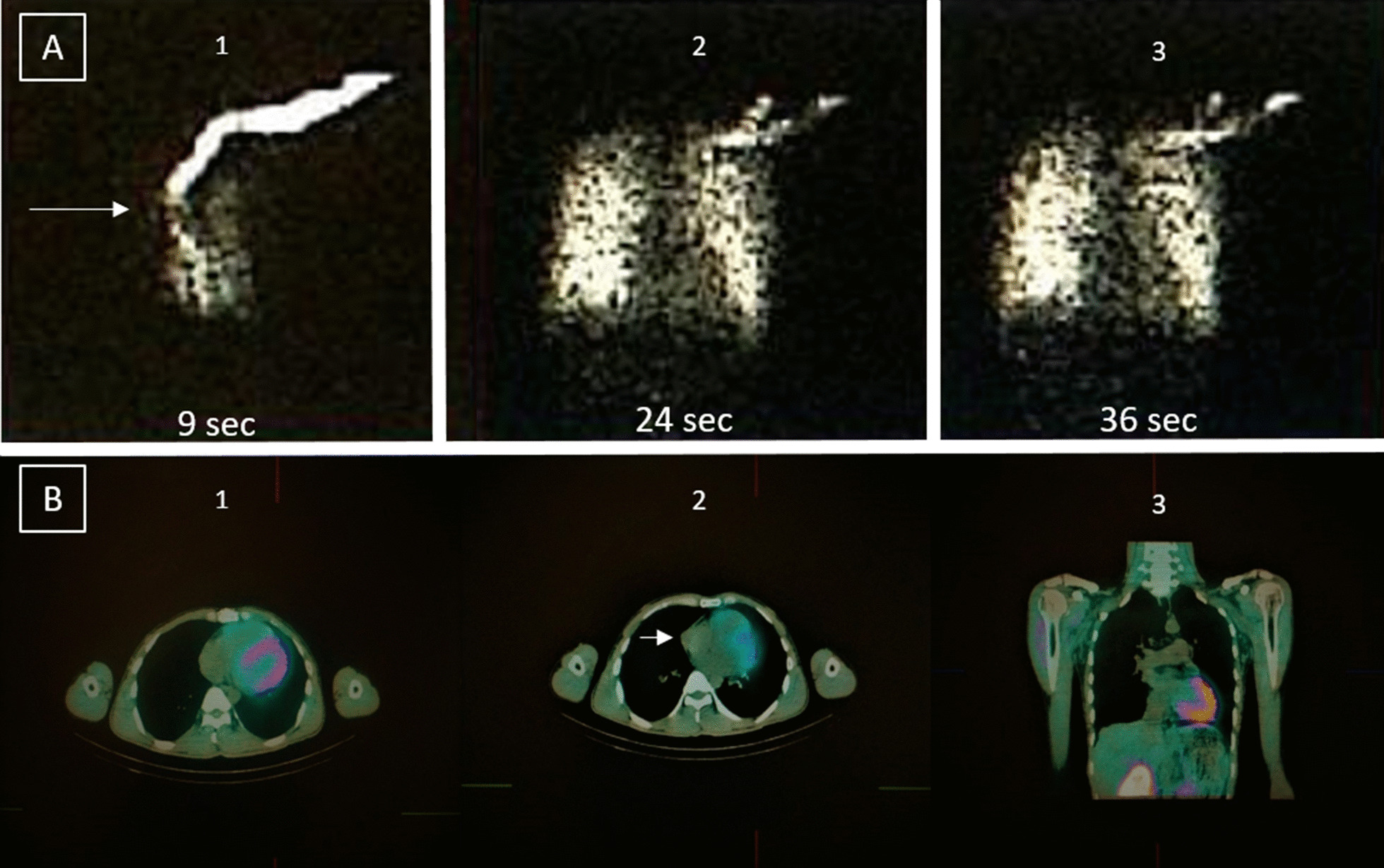
Nuclear Imaging. SPECT/CT with TC99m -MAA (**a**) and Sestamibi (**b**), filling defect (white arrow) in radioactivity in SVC before entering RA (a1); No radioactivity traced in the cardiac chamber (if there was intracardiac mass should be detected from a2). Physiologic radioactivity uptake of the myocardium (**b**), with no increased radioactivity uptake in pericardial mass near RA (b2) and RV (b3)

**Fig. 4:**
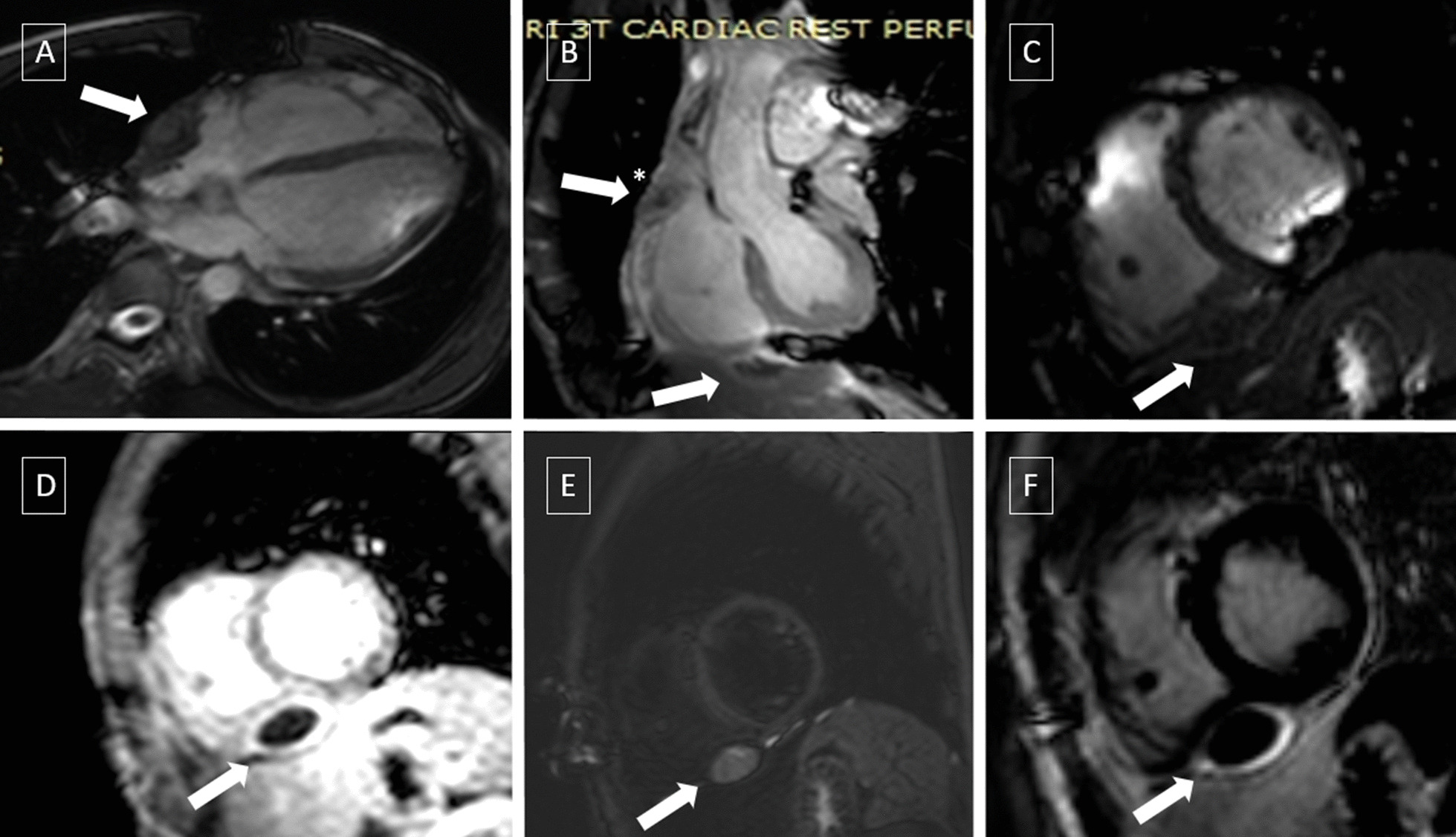
Cardiac MRI. Cardiac MRI was performed with axial sequence white blood, black blood, cine short axis, 4-chamber, 2-chamber, short axis, T2 Short Tau Inversion Recovery (STIR), Look-locker, LGE with Multihance contrast 0.5 mmol/mL, 12 mL. Balance Turbo Field Echo (BTFE) horizontal long axis 4-chambers view **a**A) showed the mass near the RA was intrapericardial. BTFE ventricular long axis focused-RV view (**b**) showed compression of superior vena cava (asterisk, *) and intrapericardial mass at RV free wall, demonstrated with BTFE—breath-hold (short-axis view) (**c**). Low T1 mapping signal on look-locker sequence (**d**), and heterogeneously high T2 signal ratio (**e**) evaluated with T2 STIR, with no evidence of gadolinium enhancement (**f**) with LGE Phase Sensitive Inversion Recovery (PSIR) confirmed the diagnosis of subacute–chronic pericardial hematoma

Single-photon emission computer tomography (SPECT)/CT with Technetium-99 m (Tc-99 m) macro-aggregated albumin (MAA) and Sestamibi was performed and did not find any radioactivity enhancement despite filling defect in the right side of the heart, which exclude intracardiac thrombus (Fig. 3). Therefore, the urgent need for anticoagulation therapy was discarded. Cardiac magnetic resonance imaging (CMRI) confirmed that the masses' location was intrapericardial (Fig. 4). Tissue characterizations imaging revealed a similar appearance of both masses, which is the dark peripheral rim and low intensity of T1 signal, heterogeneously high intensity on T2 signal with no evidence of gadolinium enhancement. The indistinct border revealed to be irregularities of the RA wall due to chronic compression. Hence, we conclude the diagnosis as multiple pericardial hematomas.

### Therapeutic intervention

We plan to observe and close follow-up because the patient was asymptomatic and hemodynamically stable.

### Follow up and outcomes

Follow-up and echocardiography were performed monthly. During the observation period, the patient's condition remained stable, and after six months, the pericardial hematomas were resolved.

## Discussion

Advancement in the field of cardiac imaging has revealed greater anatomical detail, hence improved disease evaluation. Various cardiac imaging modalities have different strengths and limitations in pericardial disease evaluation (Table 1). Echocardiography remains the first-line imaging modality in the assessment of pericardial disease [2]. Other non-invasive imaging techniques, such as CT and CMRI, allows for more accurate morphologic and physiologic evaluation. Invasive modalities such as cardiac catheterization are reserved only for hemodynamic assessment [3].

**Table 1:** Advantages and disadvantages of multi-modality  imaging in pericardial disease evaluation [3–5]

Modalities	Advantages	Disadvantages
Echocardiography	Easily performed (bedside and in emergency setting) Identification of: Pericardial effusion Intrapericardial clot Hemodynamic assessment for: Tamponade Constrictive physiology	Limitation for window: Narrow field of view Poor window in obesity, Chronic obstructive pulmonary disease (COPD), or patient with mechanical ventilation Limited in the assessment of: Pericardial thickness Tissue characterization
CT Scan	Identification of: Pericardial thickening Pericardial effusion Pericardial mass (cysts, thrombus/hematoma) Pericardial calcification Evaluation of associated intrathoracic abnormalities, such as: Pleural abnormalities (thickening, effusion) Pulmonary abnormalities (masses or other lesions) Lymph node involvement	Risks associated with radiation and contrast Unsuitable in critically-ill or uncooperative patients Limitation in: Evaluation of the elasticity of the pericardium Hemodynamic assessment, especially in assessing constrictive and tamponade physiology Differentiation of hemorrhagic effusions with thrombus/ hematoma (similar Hounsfield unit attenuation)
CMRI	A more detailed evaluation of pericardial anatomy Assessment of pericardial thickness Identification and characterizations of Pericardial fluid Pericardial masses	High cost, time-consuming Unsuitable in critically-ill or uncooperative patients Limited use in: Metal prosthetic; ICD or pacemaker-implanted patient End-stage renal disease: related to contrast Poor-quality for calcification evaluation
Nuclear imaging	Hybrid: Anatomic and metabolic evaluation Identification and detection of: Neoplasm: FDG/PET Infection/Inflammation	High cost, high maintenance Risk of radiation No data on pericardial hematoma and cyst

Pericardial mass poses a diagnostic challenge. Very limited studies published the incidence and prevalence of pericardial mass. The differential diagnosis of pericardial mass lesions includes benign, malignant primary, secondary metastatic pericardiac tumor, or non-neoplastic (Table 2) [2, 6]. The pericardial cyst is the most common pericardial mass, while pericardial hematoma is rare and only described by several case reports [7–10]. Cardiac imaging plays a crucial role in assessing pericardial mass as it has key features for each lesion (Table 3). In atypical findings, multi-modality imaging is needed to establish the diagnosis, as in our case.

**Table 2:** Differential diagnosis of pericardial mass [6, 11]

Neoplastic	
Primary	
Benign	
Malignant	
Secondary (Metastatic)	
Non-neoplastic	
Cyst	
Pericardial diverticulum	
Inflammatory pseudotumor	
Hematoma, thrombus, or clot	
Pseudoaneurysm

**Table 3:** Key radiographic findings of pericardial mass [3, 4, 6, 12–17]

	Echocardiography	CT Scan	CMR	Nuclear imaging
Pericardial cyst	Echo-lucent Located most often in the right cardio-phrenic angle No flow by color or doppler	Thin, smooth wall with no septation Attenuation between 30–40 HU No enhancement with contrast	Homogenous, unilocular, sharply marginated T1/T2 Signal: Low/High No enhancement with gadolinium	N/A
Pericardial Hematoma	Gelatinous-like appearance, distinct margins, overtime: more echo-dense	Attenuation > 60 HU for acute hematoma, which slowly decreases over time Calcification in chronic hematoma No enhancement with contrast	T1/T2 signal intensity: Acute: High/High Subacute: Heterogeneously high/high Chronic: Low/Low No enhancement with gadolinium	N/A
Pericardial neoplasm	Echo-dense Nonmobile Maybe nodular or diffuse; solitary or multiple May accompanied by effusion and thickening of the pericardium	Benign: pedunculated or sessile masses Malignant: irregular, thickened, nodular, or plaque lesions with a variable amount of effusion (mostly hemorrhagic) Variable attenuation depends on the mass type Enhancement with contrast	Heterogenous on T1 Heterogenous on T2 Mostly enhance on LGE Notes: Lipoma, Rhabdomyoma show no uptake, whereas lymphoma show no or minimal uptake	PET/CT: High FDG Uptake (mesothelioma, lymphoma)

In the pericardial cavity, echocardiography can give valuable information regarding consistency and density. Echo-lucent mass is more likely to represent fluid-filled consistencies, whereas echo-dense mass is characteristic for lesions with more solid consistencies [12]. A pericardial cyst would appear as an echo-lucent lesion, which is typically located at a cardio-phrenic angle. Meanwhile, a pericardial hematoma is generally characterized by the gelatinous-like appearance of fine-speckle echo, with distinct margins from the surrounding structure located between both pericardial layers. Previous studies have described the nature of pericardial hematoma. Initially, it appears as a more echo-lucent mass in the acute phase and becomes denser in the chronic phase, making it harder to differentiate from a solid mass [3, 13]. A neoplasm also has an echo-dense appearance similar to a chronic pericardial hematoma; nonetheless, it is usually associated with pericardial thickening and pericardial effusion [4].

As in our case, the echocardiogram findings are more suitable for multiple pericardial hematomas. However, as for the lesion near the RA, the border was still not clearly defined. Also, echocardiogram provides limited information on soft tissue characteristics [3, 14]. Hence, we made a diagnosis of intrapericardial hematoma, differential diagnosed with intracardiac thrombus. RA thrombus has similar characteristics with pericardial hematomas and could also be found as an open-heart surgery complication due to RA incision [6]. Definitive diagnosis is essential because it will affect further management; thus, we decide to proceed with additional diagnostic imaging.

Cardiac CT is superior in assessing calcified masses and global assessment of the chest and lung tissue. It also could describe the size, shape, and location of a pericardial lesion. Typically, pericardial hematoma appeared as a mass with a well-defined border. It has an attenuation of more than 60 HU in the acute phase but decreased in the chronic phase [12]. The attenuation of pericardial neoplasms is variable depending on the lesion [15]. In our case, the attenuation was around 30–40 HU for both masses, and the border in RA mass was indistinguishable, which are not typical findings for pericardial hematoma.

Furthermore, multiple mediastinal lymphadenopathies were also visualized. Mediastinal lymphadenopathy is usually associated with malignancy, but other possible causes should be considered, such as an immune response after open-heart surgery. McCarthy et al. demonstrated approximately 44% of patients with a history of open cardiac surgery had enlarged mediastinal and hilar lymph nodes [18, 19]. After consultation with the haemato-oncologists and cardiothoracic surgeons, we recommended the patient undergo an open biopsy, but he refused. Therefore, we decide to pursue the diagnosis with cardiac imaging modalities.

Nuclear imaging offered a safe, non-invasive diagnostic procedure with many advantages in evaluating cardiac' anatomical structure and function. SPECT/CT perfusion imaging is a nuclear medicine scan, which more cost-effective than the Positron Emission Tomography (PET)/CT scan; nonetheless, the PET/CT scan provides superior accuracy [20]. Both procedures are not widely used in evaluating cardiac mass, but several case reports have described several radiopharmaceuticals' applicability in intracardiac thrombus imaging [21, 22]. F-fluorodeoxyglucose PET imaging (FDG-PET) is the most established nuclear imaging for malignancy evaluation by showing hypermetabolic activity due to increased glucose metabolism in most tumor types [23, 24].

We proceed with SPECT/CT with Technetium-99 m (Tc-99 m) macro aggregated albumin (MAA) and Sestamibi scan. Previous studies have described Tc-99 m MAA's role in detecting RA thrombus and pulmonary embolism [25, 26]. Sestamibi is an established radiopharmaceutical for myocardial perfusion and malignancy evaluation, but its role in evaluating pericardial mass has never been described [27–30]. In our case, SPECT/CT showed no increase in radioactivity while filling defect was visualized. Based on the nuclear scan, intracardiac thrombus could be excluded, and the urgent for anticoagulation therapy could be discarded.

CMRI provides several advantages in imaging evaluation. It has a larger view, greater tissue contrast, and better tissue characterizations than other non-invasive modalities. The superiority of vascular and tissue characterization derived from signalling patterns with T1-/T2-weighted techniques and contrast–enhanced methods [14]. Typical findings for pericardial cyst are thin-walled fluid-filled mass, low T1–high T2 signal, without late gadolinium enhancement (LGE) [3]. The pericardial hematoma is characterized by signal intensity that changed over time, which is homogenous high T1/T2 signal for acute hematoma, heterogenous high T1/T2 signal with dark peripheral rim for subacute hematoma, and low T1 /T2 signal for chronic hematoma. Sometimes, small foci can also be detected in chronic hematoma, representing calcification, fibrosis, or hemosiderin deposition [6, 12]. Neoplasms are typically recognized by focal obliteration of the pericardial line, haemorrhagic pericardial effusion, varying the tissue's signal intensity of T1/T2, and the presence of LGE. [6, 14].

In our case, we found multiple intrapericardial mass with dark peripheral rim, low T1 signal, heterogeneously high T2 signal, and no evidence of gadolinium enhancement. These findings were consistent for subacute—chronic hematoma. Furthermore, the indistinct border was revealed as the RA wall's irregularities caused by chronic pressure by the pericardial mass (Fig. 2). Hence, we conclude the diagnosis as multiple pericardial hematomas.

Pericardial hematoma could occur after several conditions, including traumatic heart injury, post-infarction myocardial rupture, aortic dissection with intra-pericardial rupture, and open cardiac surgery. Risks of pericardial hematoma after open cardiac surgery are abnormal bleeding or coagulopathy, early use of anticoagulants, re-do surgery, valve surgery, and aortic surgery [3]. However, we did not find any of those risk factors in our patient.

Pericardial hematoma is classified as acute if less than one week, subacute if it occurs in one until four weeks, and chronic if the hematoma persistent for more than a month after the surgery. Our patient has a history of open cardiac surgery for ASD closure about one month ago, hence classified as subacute—chronic conditions. This is consistent with his imaging findings, which represent subacute characteristics.

Pericardial hematoma could regress without sequelae or progress into a larger mass. These lesions' expanding nature may be related to inflammatory reactions generated by blood's irritant effect and its breakdown products, resulting in the effusion and new bleeding from damaged micro vessels beneath the capsule [8, 31]. Small hematoma would not cause any hemodynamic consequences; however, hematoma with sufficient size could press surrounding structures, such as great vessels and chambers, leading to compression syndrome [3, 31]. In our case, the pericardial hematomas were resolved within six months.

Based on our knowledge, this is the first case report of multiple pericardial hematomas post-cardiac surgery. Due to limited cases, there is no uniformity in the diagnostic approach and its management. In stable patients, as in our case, we choose to perform diagnostic investigation and monitoring for the disease progression meticulously. Several case reports have described the outcome of the pericardial hematoma, in which most cases lead to hemodynamic instability leading to emergency surgery or death [7–9].

## Conclusion

Physicians should consider multiple pericardial hematomas as a cause of pericardial masses after cardiac surgery. Sometimes, multi-modality cardiac imaging is needed to establish the diagnosis, particularly in patient with atypical imaging findings. Careful and meticulous follow-up should be considered for an asymptomatic patient with stable hemodynamic.

## Data Availability

The datasets used and/or analysed during the current study are available from the corresponding author on reasonable request.

## Abbreviations

ASD: Atrial septal defect
BTFE: Balance turbo field echo
CMRI: Cardiac magnetic resonance imaging
COPD: Chronic obstructive pulmonary disease
CT: Computed tomography
ECG: Electrocardiography
FDG: F-fluorodeoxyglucose
HU: Hounsfield unit
LGE: Late gadolinium enhancement
MAA: Macro-aggregated albumin
MPR: Multi planar reconstructions
PET: Positron emission tomography
PSIR: Phase sensitive inversion recovery
RA: Right atrium
RV: Right ventricle
SPECT: Single-photon emission computer tomography
STIR: Short tau inversion recovery
Tc-99 m: Technetium-99 m
TEE: Trans-oesophageal echocardiography
TTE: Transthoracic echocardiography

## References

[1] Tower-Rader A, Kwon D (2017). Pericardial masses, cysts and diverticula: a comprehensive review using multi-modality imaging. Prog Cardiovasc Dis.

[2] Restrepo CS, Vargas D, Ocazionez D, Martínez-Jiménez S, Betancourt Cuellar SL, Gutierrez FR (2013). Primary pericardial tumors. Radiographics.

[3] Hutchison SJ (2009). Pericardial diseases: clinical diagnostic imaging atlas with DVD.

[4] Klein AL, Abbara S, Agler DA, Appleton CP, Asher CR, Hoit B, et al. American Society of Echocardiography clinical recommendations for multi-modality cardiovascular imaging of patients with pericardial disease: endorsed by the Society for Cardiovascular Magnetic Resonance and Society of Cardiovascular Computed Tomography. J Am Soc Echocardiogr. 2013;26(9):965–1012. e15. 10.1016/j.echo.2013.06.023 23998693

[5] Delbeke D, Schöder H, Martin WH, Wahl RL, editors. Hybrid imaging (SPECT/CT and PET/CT): improving therapeutic decisions. Seminars in nuclear medicine; 2009: Elsevier. 10.1053/j.semnuclmed.2009.03.002 19646557

[6] Wang ZJ, Reddy GP, Gotway MB, Yeh BM, Hetts SW, Higgins CB (2003). CT and MR imaging of pericardial disease. Radiographics.

[7] Sridhara S, Lichtenwalter C, Mazdeh S, Yeneneh BT (2018). Positive Murphy’s sign in pericardial hematoma from a right atrial tear. Cureus.

[8] Fernandes A, Cassandra M, Pinto C, Oliveira C, Antunes M, Gonçalves L. Loculated cardiac hematoma causing hemodynamic compromise after cardiac surgery. Revista Portuguesa de Cardiologia. 2015;34(9):561. e1-e3. 10.1016/j.repc.2015.01.020 26300161

[9] Huang HD, Garcia M, Alam M, Misra A, Lakkis N, Tabbaa R (2014). Post-operative intrapericardial hematoma presenting as isolated right atrial tamponade. J Cardiol Cases.

[10] Ortega JR, San Román JA, Rollán MJ, García A, Tejedor P, Huerta R (2002). Atrial hematoma in cardiac postoperative patients and the diagnostic use of transesophageal echocardiography. Rev Esp Cardiol.

[11] Zhou W, Srichai MB (2017). Multi-modality imaging assessment of pericardial masses. Curr Cardiol Rep.

[12] Herzog E (2014). Management of pericardial disease.

[13] Fyke FE, Tancredi RG, Shub C, Julsrud PR, Sheedy PF (1985). Detection of intrapericardial hematoma after open heart surgery: the roles of echocardiography and computed tomography. J Am Coll Cardiol.

[14] Motwani M, Kidambi A, Herzog BA, Uddin A, Greenwood JP, Plein S (2013). MR imaging of cardiac tumors and masses: a review of methods and clinical applications. Radiology.

[15] Lilly LS, Braunwald E (2012). Braunwald's heart disease: a textbook of cardiovascular medicine.

[16] Makis W, Ciarallo A, Hickeson M, Rush C, Novales-Diaz JA, Derbekyan V, et al. FDG PET/CT. 2011.

[17] Makis W, Ciarallo A, Hickeson M, Rush C, Novales-Diaz JA, Derbekyan V (2012). Spectrum of malignant pleural and pericardial disease on FDG PET/CT. Am J Roentgenol.

[18] McCarthy CJ, O'Neill AC, McEvoy SH, Kilcoyne A, Butler MW, Keane MP (2013). Poststernotomy lymphadenopathy: prevalence, size, and location on chest CT. Diagnostic Intervent Radiol (Ankara, Turkey).

[19] McCarthy A, Mohan S, Saeed A, Kumaran M (2014). Mediastinal lymphadenopathy, a review of nodal anatomy, pathology and sampling techniques. Imaging.

[20] Bateman TM (2012). Advantages and disadvantages of PET and SPECT in a busy clinical practice. J Nucl Cardiol.

[21] Zanglis A, Andreopoulos D, Baziotis N (2007). Trapping of technetium-99m albumin macroaggregate and other four radiopharmaceuticals by blood clots in vitro. Hell J Nucl Med.

[22] Nishimura T, Misawa T, Park Y-D, Uehara T, Hayashida K, Hayashi M (1987). Visualization of right atrial thrombus associated with constrictive pericarditis by indium-111 oxine platelet imaging. J Nucl Med.

[23] Restrepo CS, Vargas D, Ocazionez D, Martínez-Jiménez S, Cuellar SLB, Gutierrez FR (2013). Primary pericardial tumors. Radiographics.

[24] Zhu A, Lee D, Shim H (2011). Metabolic positron emission tomography imaging in cancer detection and therapy response. Semin Oncol.

[25] Zanglis A, Andreopoulos D, Dima M, Sidiropoulou A, Baziotis N (2006). A large intra-atrial thrombus detected during a lung perfusion scan with technetium-99m macroaggregated albumin injected through the subclavian venous line. Hell J Nucl Med.

[26] Gandhi SJ, Babu S, Subramanyam P, Shanmuga SP (2013). Tc-99m macro aggregated albumin scintigraphy—indications other than pulmonary embolism: a pictorial essay. Indian J Nuclear Med IJNM.

[27] Reyhan M, Aydin M, Yapar AF, Bolat FA, Tercan F (2004). Atypical carcinoid tumor detected incidentally on Tc-99m sestamibi myocardial perfusion scintigraphy. Clin Nucl Med.

[28] Desai SP, Yuille DL (1993). Visualization of a recurrent carcinoid tumor and an occult distant metastasis by technetium-99m-sestamibi. J Nuclear Med.

[29] Maffioli L, Steens J, Pauwels E, Bombardieri E (1996). Applications of 99mTc-sestamibi in oncology. Tumori J.

[30] Dvorak RA, Brown RKJ, Corbett JR (2011). Interpretation of SPECT/CT myocardial perfusion images: common artifacts and quality control techniques. Radiographics.

[31] Hirai S, Hamanaka Y, Mitsui N, Isaka M, Kobayashi T (2003). Chronic expanding hematoma in the pericardial cavity after cardiac surgery. Ann Thorac Surg.

